# Surgeon Health and Well-Being Initiative: A Survey of General and Orthopedic Surgery Residents' Reports of Musculoskeletal Pain

**DOI:** 10.7759/cureus.115174

**Published:** 2026-08-25

**Authors:** Maryam Mohamed, Grace J Kim, Kristen Conrad-Schnetz, Zhan Yang, Mary Carneval

**Affiliations:** 1 Anesthesiology, OhioHealth, Cleveland, USA; 2 General Surgery, Cleveland Clinic South Pointe Hospital, Cleveland, USA; 3 Wellness and Integrative Medicine, Cleveland Clinic South Pointe Hospital, Cleveland, USA; 4 General Surgery, Cleveland Clinic Euclid Hospital, Cleveland, USA; 5 Surgery, Cleveland Clinic Lerner College of Medicine, Case Western Reserve University School of Medicine, Cleveland, USA; 6 General Surgery, Ohio University Heritage College of Osteopathic Medicine, Cleveland, USA

**Keywords:** career longevity, disability, general surgeon, general surgery residents, musculoskeletal pain, occupational pain, orthopedic surgery residents, osteopathic manipulative treatment (omt), surgeon wellness, surgical ergonomics

## Abstract

Background

Musculoskeletal (MSK) pain is a well-known risk among surgeons, resulting from the multifactorial physical demands of the occupation. Because of the demands faced by resident surgeons, many individuals find themselves with sometimes debilitating MSK pain. Although these risks are well documented and there have been proposed solutions, there is little discussion on the role that osteopathic manipulative treatment (OMT) can play in relief of MSK pain over time in this population.

Objective

The purpose of this study is to determine the prevalence and distribution of MSK pain among general and orthopedic surgery residents at Cleveland Clinic Foundation (CCF). Based on the results, future work incorporating OMT during surgical residency could be done to address both the physical and psychosocial aspects of their pain.

Methodology

From April 10, 2024, through June 30, 2024, an observational survey study was conducted among general and orthopedic surgery residents at CCF. An online survey was sent to 164 residents. To maximize the response rate during that time period, three follow-up emails were sent. The survey asked residents: postgraduate year (PGY), gender, age group, whether they have completed a research year, any past injuries, and how often they experienced pain at various anatomical regions.

Results

A total of 43 of 164 (26.2%) CCF orthopedic and general surgery residents responded to the survey: 20 (46.5%) men and 23 (53.5%) women. The mean age of participants was 30-34 years. The PGY level ranged from 1 to 7, with 16/43 (37.2%) participants having completed a research year. Among the respondents of the online survey, at least one resident reported pain in all regions listed on the survey. The three most common were neck pain in 35/43 (81.3%), lower back pain in 34/43 (79.0%), and upper back pain in 25/43 (58.1%) of participants.

Conclusions

Our study demonstrates that daily to weekly MSK pain starts in residency as early as PGY 1, with the mean age of residents surveyed being 30-34 years old. This is regardless of past injuries. MSK pain is a serious consequence of the occupational hazards of the surgical field. Our study demonstrates that the neck, lower back, and the upper back are the three most common anatomical regions where CCF general and orthopedic surgery residents experience MSK pain. Prior research focuses on MSK pain throughout a surgeon’s career. Additional research is needed to better characterize the cause of MSK pain in surgical residents and to evaluate OMT as a potential treatment intervention for this population.

## Introduction

What comes with a surgical career is the willingness to engage in a physically demanding surgical practice. The demands begin as early as medical school, when students are doing their required third-year surgical rotation, and continue for decades if a surgical specialty is pursued. The residency years should not be ignored when it comes to physical demand and stamina. In the past several years, attention has been given to surgical ergonomics and its importance. This includes the creation of ergonomically correct surgical instruments and coaching for optimal posture during surgical procedures. Maintenance of physical fitness and recovery after physical strain are of utmost importance, as well as treatment of musculoskeletal (MSK) pain that develops. 

MSK pain is a debilitating condition in the surgical realm, causing challenges to surgeon performance, endurance, and well-being. With an environment constantly demanding prolonged periods of standing, stagnant positions that are often required to be held for long periods of time, repetitive movements, and ergonomic challenges, surgeons are at high risk for MSK pain [1]. Stucky et al. reported a 68% prevalence of generalized pain amongst surveyed surgeons, especially in the neck, back, and arm/shoulder [2]. This pain was more likely to be present in surgeons who performed mainly laparoscopic surgery, which often requires strained and still positions. These results were echoed in a study reporting an 87% prevalence of MSK pain amongst surgeons who regularly perform laparoscopic surgeries [3,4].

Pain is the signal of injury to the body. Chronic pain can eventually progress into MSK disorders with lasting or permanent damage. In another meta-analysis, Epstein et al. reported a 35%-60% prevalence and found that the most common MSK disorders included cervical and lumbar spine degeneration, rotator cuff injury, and carpal tunnel syndrome [5]. However, the prevalence of pain is as high as 97% [6]. In addition to this, those with an MSK disorder had a 12% chance of being forced into early retirement, leave of absence, or practice restriction [5]. Both general surgery and orthopedic surgery were two of twelve specialties that were identified as at-risk specialties for their lack of awareness of ergonomic recommendations for the operating room. Some studies included in the meta-analysis found that teaching medical trainees operating ergonomics was feasible and affected a change in behavior and reduced symptoms [7].

Shah et al. surveyed surgical attendings and residents at a single institution and found that most participants reported MSK pain. In addition, 76.4% of participants required treatments for their MSK pain in the form of over-the-counter medications, prescription pain medications, physical therapy, orthopedic surgery, and/or additional testing, such as labs and imaging. Surprisingly, residents and attendings had similar rates not only of requiring treatment, but also of modality of treatment, suggesting that MSK pain starts early in one’s surgical career [8].

Further complicating the issue, only about 20% of occupation-related injuries for surgeons were reported to their institutions despite surgeons taking days off and performing fewer surgeries due to injury and pain, suggesting that rates of MSK pain and injury are vastly underestimated [9].

The purpose of this paper is to identify any patterns of MSK pain in general and orthopedic surgery residents so that a case can be made to offer voluntary interventions such as being evaluated and treated with OMT throughout the residency years. We hypothesize that residents are experiencing MSK pain during their residency training years and that there are more reports of pain in older residents as well as those who have experienced injuries before. 

To date, there are no formally established or studied OMT programs for surgical trainees to treat their pain. Based on these results, ongoing research and development of surgical trainee physical wellness programs, which could include OMT, can be established. 

This article was previously presented as a poster at the 2025 Ohio Osteopathic Association Symposium Research and Scholarly Activity Competition on April 12, 2025.

## Materials and methods

This observational study was reviewed and approved by the Institutional Review Board of the Cleveland Clinic Foundation (CCF) (IRB #24-087). A voluntary online survey was sent to 164 categorical general and orthopedic surgery residents at Cleveland Clinic Main Campus, Cleveland Clinic South Pointe, and Cleveland Clinic Akron General hospitals starting on April 10, 2024, and ending on June 30, 2024. For this project, we chose to begin the query with surgical residencies at the hospital where the authors (MC, KC, ZY) serve as faculty, of which there are two: orthopedic surgery and general surgery. Considering the small number of residents between the two programs at that hospital, the inclusion criteria were expanded to include orthopedic and general surgery residents from programs at two additional in-system hospital programs and kept to these specialties for the sake of uniformity. The program directors for each of the six programs gave approval to survey the residents in the programs.

The survey sent to residents was adapted from the Cornell Musculoskeletal Discomfort Questionnaire (CMDQ), a freely available screening tool, developed by the Human Factors and Ergonomics Laboratory at Cornell University, which has been internationally validated for both sedentary and standing workers [10-13]. Written permission to use the instrument was granted by Dr. Alan Hedge, the developer of the questionnaire. Our survey was adapted from the CMDQ for standing workers and constructed on a REDCap survey form. The survey sent to residents is provided in the Appendix. The survey asks how often participants experience pain at various anatomical regions. Frequency of pain experienced was split into five categories: never, 1-2 times last week, 3-4 times last week, once every day, or several times every day. A personalized survey link was sent to each resident, being explicit about the optional nature of the survey, and results were collected in a REDCap database to maintain data security and anonymity. Participants were made aware that the survey was completely voluntary, with no penalty for withdrawing. Consent was implied by completion of the study. Demographic information was also obtained, including PGY, residency program, sex, age group, whether they have completed a research year, and any past injuries. The residents were sent follow-up emails reminding them to complete the surveys. Residents were incentivized to complete the survey with a $10 gift card. The residents’ identifying information was voluntarily recorded if the participants opted to collect gift cards. Funding was secured through the Brentwood Foundation, a non-profit, local organization.

Statistical analysis

Initial statistical analysis was carried out by a statistician with Ohio University Heritage College of Osteopathic Medicine (OU-HCOM), where an author (MM) attended medical school. Two authors (MM and GK) then used descriptive statistics (e.g., means, mode, percentages) to further characterize the three most common areas of MSK pain as reported in the survey adapted from the CMDQ.

## Results

From April 10, 2024, to June 20, 2024, an online survey was sent to 164 general surgery and orthopedic surgery residents at the CCF. A total of 43 residents responded to the survey, including 20 (46.5%) men and 23 (53.5%) women. The mean age of participants was 30-34 years. The PGY levels ranged from 1 to 7, with 9 (20.9%) participants in PGY 1, 9 (20.9%) in PGY 2, 8 (18.6%) in PGY 3, 9 (20.9%) in PGY 4, 5 (11.6%) in PGY 5, 2 (4.6%) in PGY 6, and 1 (2.3%) in PGY 7. The percentage of participants who had completed a research year was 16 (37.2%) (Table 1).

**Table 1:** Survey participant demographics. Total number of participants: 43. PGY, postgraduate year

Category	Value
Total participants	43
Male	20 (46.5%)
Female	23 (53.5%)
PGY 1	9 (20.9%)
PGY 2	9 (20.9%)
PGY 3	8 (18.6%)
PGY 4	9 (20.9%)
PGY 5	5 (11.6%)
PGY 6	2 (4.6%)
PGY 7	1 (2.3%)
Past injuries	
None	23 (53.5%)
Yes	20 (46.5%)
Age (years)	
25-29	15 (34.8%)
30-34	25 (58.1%)
35-39	3 (6.9%)

**Figure 1:**
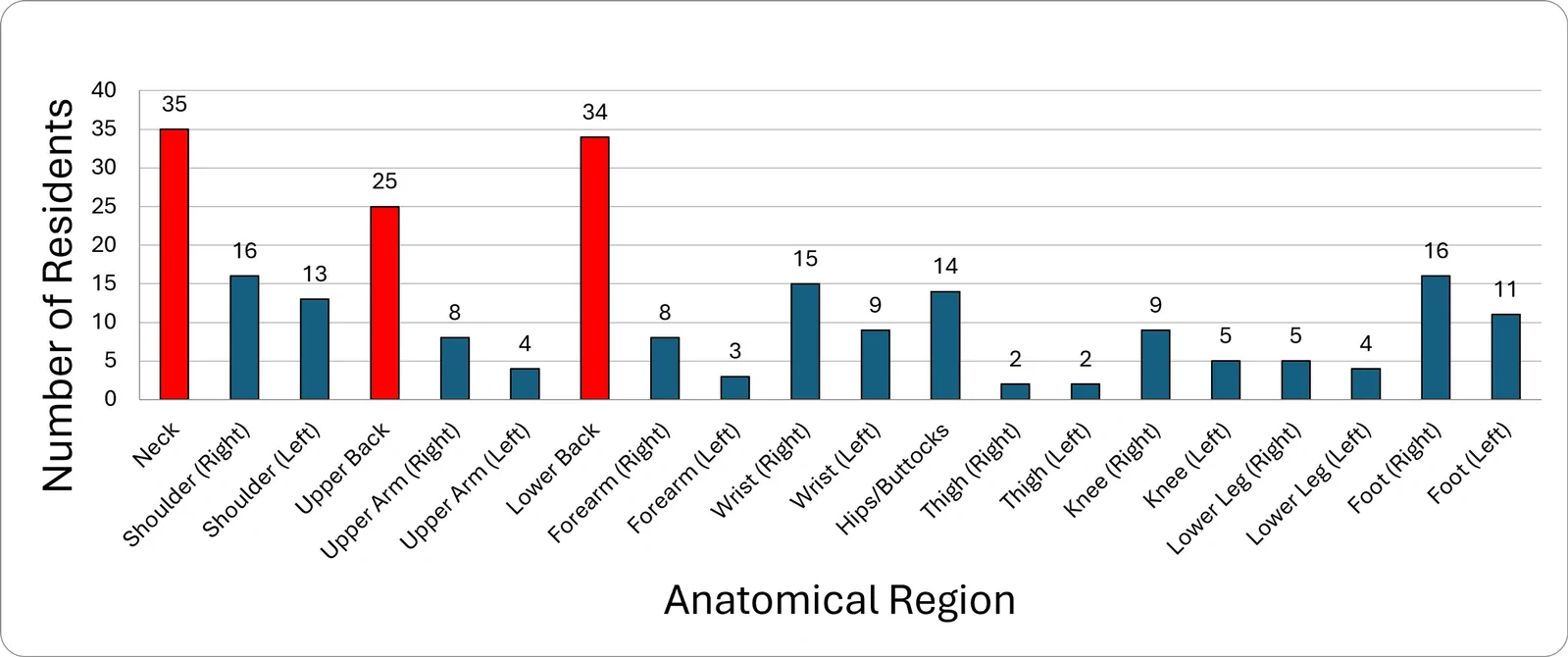
Anatomical regions and the number of residents who reported pain in those regions. Total number of participants: 43.

Among the 43 survey respondents, the three most commonly reported areas of pain were the neck in 35 (81.3%), lower back in 34 (79%), and upper back in 25 (58.1%) of participants (Figure 1).

As for frequency, neck pain was reported to occur one to two times a week in 18 (51.4%) participants and three to four times a week in 10 (28.6%) participants. Regarding the number of residents reporting neck pain, all PGY 5 (5/5, 100.0%), PGY 6 (2/2, 100.0%), and PGY 7 (1/1, 100.0%) residents reported pain in this region. High proportions were observed among lower PGY levels: PGY 4, 8 (88.9%); PGY 3, 6 (75.0%); PGY 2, 6 (66.7%); and PGY 1, 7 (77.8%). Three (100.0%) residents aged 35-39 years reported neck pain, while 22 (88.0%) of those aged 30-34 years experienced this pain. Although 10 of 15 surveyed residents aged 25-29 years reported lower daily or weekly rates of neck pain, the overall rate was still over 65.0%.

For upper back pain, 19 (88.9%) PGY 1 residents reported the highest rates, and 16 (64.0%) experienced pain one to two times a week. PGY 5 was the next most frequent, with five (80.0%) experiencing pain one to two times a week. Eight (62.5%) PGY 3 residents also reported high rates of upper back pain. Nine (33.3%) PGY 2 and nine (33.2%) PGY 4 residents reported the lowest rates. The prevalence of upper back pain was highest among 16 (64.0%) of those aged 30-34 years, compared with eight (53.3%) of those who were younger and one (33.3%) of those who were older.

For low back pain, nine (88.9%) PGY 1 residents reported pain in this region. Twelve (35.3%) residents stated that they experienced low back pain one to two times a week, and 11 (31.4%) residents experienced it three to four times a week. PGY 5 was the next most frequent year, with five (80%) participants reporting low back pain. Nine (77.8%) PGY 2 and nine (77.8%) PGY 4 residents reported low back pain at the same rate, and eight (75.0%) PGY 3 residents reported low back pain. Two (50.0%) PGY 6 residents stated that they experienced low back pain. In terms of age, 13 (86.7%) participants in the 25-29 year age group reported low back pain.

**Figure 2:**
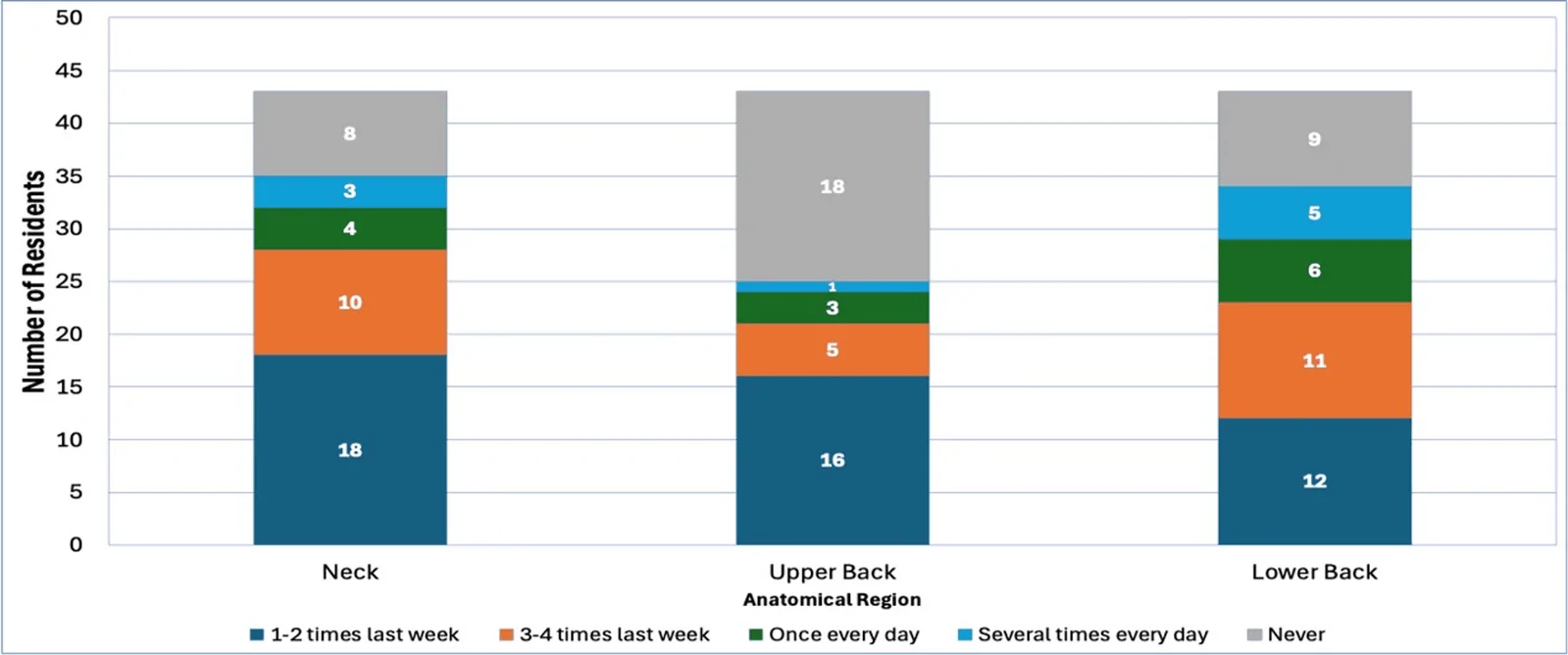
Graph depicting the top three regions of pain and the frequency that all surveyed residents reported experiencing. Total number of participants: 43.

As mentioned earlier, this study measured the severity of pain by asking residents how often they experience pain in different anatomical regions. This was split into four categories: never, one to two times last week, three to four times last week, once every day, and several times every day (Figure 2).

The percentage of participants reporting pain in other areas was as follows: 16 (37.2%) reported right shoulder pain, 13 (30.2%) left shoulder pain, eight (18.6%) right upper arm pain, four (9.3%) left upper arm pain, eight (18.6%) right forearm pain, three (6.9%) left forearm pain, nine (20.9%) left wrist pain, 14 (30.2%) hip/buttock pain, two (4.6%) right thigh pain, two (4.6%) left thigh pain, nine (20.9%) right knee pain, five (11.6%) left knee pain, five (11.6%) right lower leg pain, four (9.3%) left lower leg pain, 16 (37.2%) right foot pain, and 11 (25.5%) left foot pain.

Overall, most residents who admitted to having neck, upper back, and lower back pain did not report having past injuries.

## Discussion

In our study, the three most commonly reported anatomic regions of pain among general and orthopedic surgery residents were the neck (35/43, 81.3%), lower back (34/43, 79.0%), and upper back (25/43, 58.1%). With respect to anatomical regions, these findings are consistent with a previous study by McQuivey et al. that observed surgical ergonomics and MSK pain in orthopedic resident physicians. This was a multicenter survey in which orthopedic surgery residents responded to an online survey, with one of the sections being symptom by body part. The two most common locations of MSK pain reported by residents in that study were lower back and neck [6]. The question then becomes why these two anatomical regions have become the most reported by residents for MSK pain. Possible contributing factors for pain in the neck and back are poor ergonomics from equipment commonly used by surgeons, including use of endoscopy, surgical loupes, head lamps, or even protective equipment, such as lead gowns and exhaust suits [6,14]. Another cause of MSK pain in the neck, lower back, and upper back is poor posture and positioning, prolonged static motion, standing for extended periods of time, repetitive movements, and high physical and mental demands [6,15].

Our study also explored whether or not past injuries had an impact on MSK pain. We looked at the three most commonly reported anatomical regions: the neck, lower back, and upper back. In terms of neck pain, of the 35 residents who experienced some frequency of pain (81.3%), 16 (45.7%) reported a past injury, whereas 19 (54.2%) reported no past injury. In terms of lower back pain, of the 34 residents who experienced some frequency of pain, 17 (50.0%) reported a past injury, whereas 17 (50.0%) reported no past injury. For upper back pain, of the 25 (58.1%) residents who reported some frequency of pain, 11 (44.0%) reported a past injury, whereas 14 (56.0%) reported no past injury. Therefore, our data suggest that there is no correlation between past injuries and MSK pain in these regions. This suggests that pain in these anatomical regions can potentially be attributed to the physical demands of surgical residency as opposed to non-occupational, chronic pain.

In terms of frequency of pain, we measured how frequently each resident experienced pain. Of those with neck pain, seven (20.0%) respondents reported experiencing pain daily. Additionally, 11 (32.3%) respondents with low back pain experienced pain daily. For upper back pain, four (16.0%) respondents experienced pain daily.

With the median age of participants in this study being 30-34 years, it is clear that MSK pain in surgeons can affect daily life and place strain on a physician’s body even at a younger age, potentially impacting performance, health, and overall well-being. Comparing this data to a study done on Mohs surgeons from Mayo Clinic in which the average age of symptom onset was 35.4 years, our population’s symptom onset is slightly younger [16]. In our study, MSK pain is highly prevalent at PGY 1 and remains consistently high throughout training. In the survey results gathered in a study conducted by Soueid et al., nearly 40% of respondents reported that work-related pain was severe enough to cause them to take a break from the procedure to provide some relief [17]. Therefore, it is imperative to begin addressing MSK pain throughout a surgeon’s career to prevent the downstream effects of chronic pain on productivity and overall well-being.

Limitations of this study are mainly in the small sample size with a response rate of 26.2%. In addition, future research would require a clearer delineation in PGY designation, as some programs are five-year programs and some are longer due to research years. Without this delineation, conclusions about correlations between MSK pain and operating time are difficult to assess. Lastly, pain was reported across residents both with and without a history of prior injuries, though the cross-sectional design precludes causal conclusions.

Osteopathic manipulative treatment (OMT) represents a promising adjunctive therapy for addressing musculoskeletal pain in US surgeons. Grounded in osteopathic principles of musculoskeletal health and whole-body integration, OMT employs a diverse array of hands-on techniques to restore musculoskeletal balance, alleviate pain, and enhance functional mobility. By targeting somatic dysfunction, fascial restriction, and neurophysiological imbalances, OMT addresses the underlying biomechanical contributors to musculoskeletal pain, offering a holistic approach to pain management and rehabilitation. Several proven techniques have been used on patients to improve their chronic pain. A randomized controlled trial performed by Groisman et al. compared pain levels of patients with non-specific neck pain [18]. One group of patients was treated with exercise alone; the other group of patients was treated with exercise and OMT. The data analysis from this study found a significant reduction in pain and improvement in functionality in both groups. In another observational study by Nicodemus et al., OMT significantly reduced pain and psychosocial features secondary to chronic lower back pain [19]. Treatments such as myofascial release, facilitated positional release, and muscle energy techniques can relieve muscle tension and reduce pain in areas of discomfort. Other techniques such as high-velocity, low-amplitude and balanced ligamentous tension methods can improve joint mobility, resulting in not only improved pain, but better range of motion to assist in surgical dexterity. Additionally, the holistic approach implemented in OMT can help reduce stress and positively increase biopsychosocial effects, leading to an overall impact on one’s musculoskeletal system and reduction in pain [19]. Further studies and research looking at OMT effectiveness and improvement of MSK pain in surgeons are needed.

## Conclusions

MSK pain is a serious consequence of the occupational hazards of the surgical field. Our study demonstrates that the neck, lower back, and upper back are the three most common anatomical regions where CCF general and orthopedic surgery residents experience MSK pain. Prior research focuses on MSK pain throughout a surgeon’s career. However, our study demonstrates that daily to weekly MSK pain starts in residency as early as PGY 1, with a mean age of 30-34 years. Although this does not demonstrate that the oldest members of the studied cohort are experiencing more pain, the fact that the average-aged respondent is experiencing MSK pain that could impact their present or future surgical career is concerning. This is regardless of past injuries. However, additional research is needed to better characterize the cause of MSK pain in surgical residencies and how to address the pain, including OMT as a possible avenue for treatment.

## Figures and Tables

**Figure 3:**
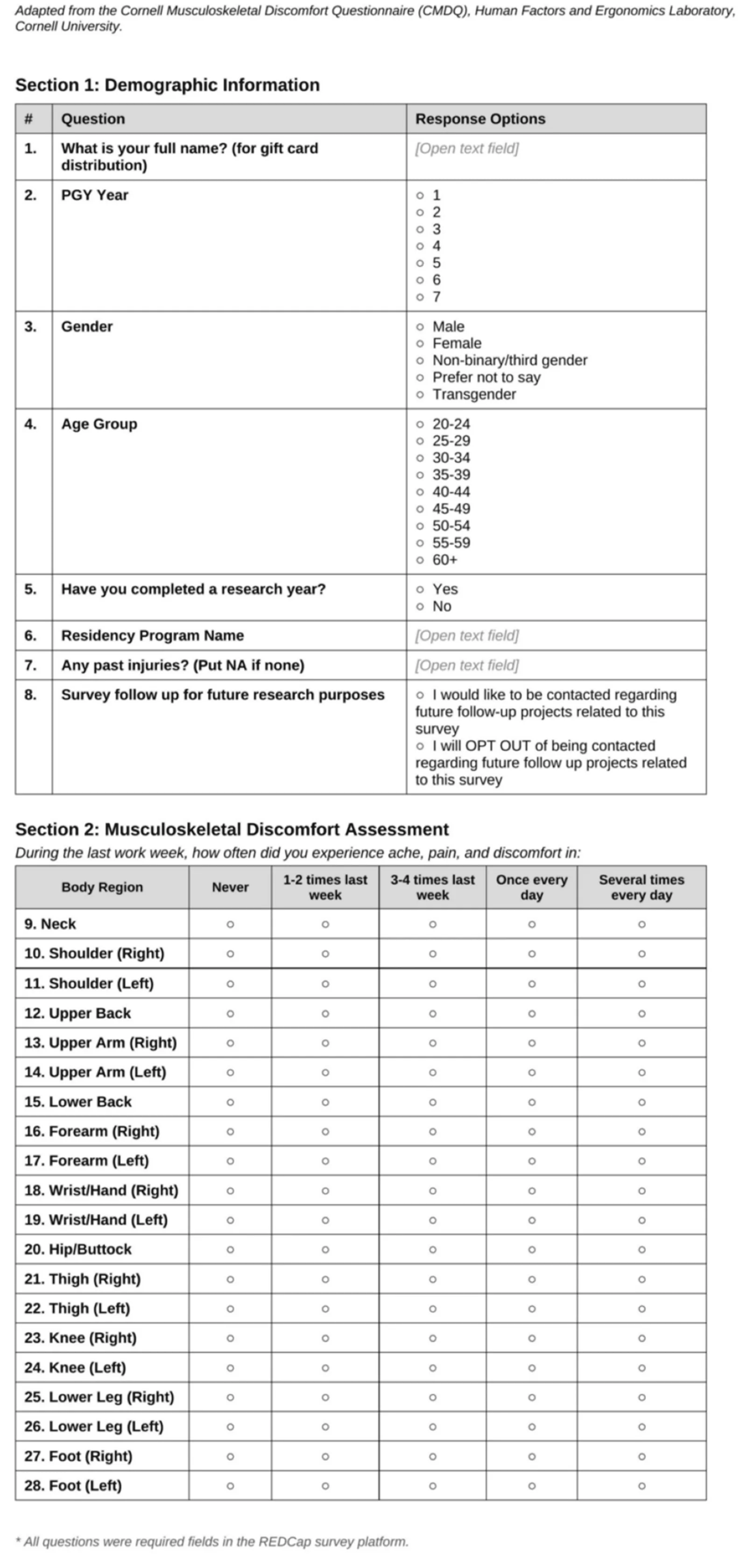
Survey sent to general and orthopedic surgery residents via REDCap. Adapted from the Cornell Musculoskeletal Discomfort Questionnaire (CMDQ), standing worker [10-13]. Written permission to use the instrument was granted by Dr. Alan Hedge, the developer of the questionnaire.

## Appendices

Appendix: Occupational pain in surgery residents survey
